# Clinicopathological Machine Learning Models for Mortality Risk Prediction in Head and Neck Squamous Cell Carcinoma: A Retrospective Analysis of the The Cancer Genome Atlas Head-Neck Squamous Cell Carcinoma (TCGA-HNSC) Cohort

**DOI:** 10.7759/cureus.116022

**Published:** 2026-09-09

**Authors:** Sharjeel Chaudhry, Zarmina Ehtesham, Fatimah AL- Ayiedh, Bader H Alqurouni, Fahad D Almutairi

**Affiliations:** 1 Molecular Pathology, Dow University of Health Sciences, Karachi, PAK; 2 Laboratory Medicine, King Fahd Military Medical Complex, Dhahran, SAU

**Keywords:** ai and machine learning, cox regression, head and neck squamous cell carcinoma (hnscc), survival analysis, tcga, the cancer genome atlas head-neck squamous cell carcinoma

## Abstract

Background: Head and neck squamous cell carcinoma (HNSCC) is a leading contributor to global cancer mortality, yet outcome prediction in routine practice still relies almost entirely on anatomical staging. We evaluated whether readily available clinicopathological variables, and their combination with tumour transcriptomic data, can support machine learning (ML)-based mortality risk stratification in HNSCC.

Methods: We analysed clinical records for 528 patients from the Cancer Genome Atlas Head-Neck Squamous Cell Carcinoma (TCGA-HNSC) cohort, obtained from the National Cancer Institute's (NCI's) Genomic Data Commons (GDC). Ten clinicopathological features were used to train four classifiers: logistic regression, random forest, gradient boosting, and support vector machine to predict vital status, evaluated by stratified five-fold cross-validation. Time-to-event outcomes were separately modelled with Kaplan-Meier estimation and multivariable Cox proportional hazards regression. In a matched subset of 500 patients with available tumour RNA-sequencing data, we additionally incorporated expression of four HNSCC driver genes (CDKN2A, EGFR, FAT1, and E2F1) identified from the TCGA landmark genomic analysis, together with the first five principal components of a 4,465-gene expression panel, and re-evaluated the same four classifiers on this matched cohort with and without the genomic features.

Results: The cohort included 224 deaths (42.4%). Using clinicopathological variables alone, random forest achieved the highest discrimination (area under the curve (AUC) 0.698), followed by logistic regression (AUC 0.689), support vector machine (AUC 0.686), and gradient boosting (AUC 0.666). Kaplan-Meier analysis showed clear separation of overall survival by stage (log-rank Stage I vs. IV, p = 0.005), and multivariable Cox regression confirmed Stage IV disease (HR 4.07, 95% CI 1.50-11.03, p = 0.006) and older age (HR 1.02 per year, p = 0.005) as independent predictors of mortality (concordance index 0.608). Adding genomic features in the matched 500-patient cohort improved discrimination for every classifier, most notably random forest (AUC 0.708 → 0.727) and logistic regression (AUC 0.688 → 0.704); expression-derived features accounted for eight of the 15 most important predictors in the augmented model.

Conclusions: Routinely collected clinicopathological data provide a modest but genuine and statistically robust prognostic signal in HNSCC (AUC 0.67-0.70). Supplementing this with a small panel of driver-gene expression values and an unsupervised transcriptomic signature further improves discrimination (best AUC 0.727) without resolving the underlying prognostic ceiling, supporting stepwise, transparently benchmarked multi-omic model development rather than reliance on any single data modality.

## Introduction

Head and neck cancer (HNC) remains one of the most common malignancies worldwide. Using Global Cancer Observatory (GLOBOCAN) 2022 and Global Burden of Disease 2021 data, Zhang et al. estimated 1,750,871 new HNC cases and 527,226 deaths among adults aged 20 years and older globally in 2022, with lip, oral cavity, and pharyngeal subsites carrying the highest age-standardised mortality [1]. Broader GLOBOCAN estimates place head and neck cancer among the leading causes of cancer death worldwide, with squamous cell histology accounting for the large majority of cases [2].

Prognosis in head and neck squamous cell carcinoma (HNSCC) is conventionally estimated using the American Joint Committee on Cancer (AJCC)/Union for International Cancer Control (UICC) TNM (Tumour, Node, Metastasis) staging system [3], which, while clinically indispensable, groups biologically heterogeneous tumours into broad categories and leaves considerable outcome variability unexplained even within a given stage; external validation cohorts have likewise shown that successive AJCC staging revisions improve, but do not eliminate, this residual prognostic heterogeneity [4]. The Cancer Genome Atlas (TCGA)-HNSCC project characterised the genomic landscape of 279 tumours. It demonstrated substantial molecular heterogeneity linked to human papillomavirus (HPV) status, smoking history, and anatomical subsite [5], reinforcing the idea that stage alone is an incomplete prognostic tool.

Machine learning (ML) offers a complementary approach: rather than relying on a single ordinal staging variable, ML classifiers can integrate multiple correlated and non-linear clinicopathological features simultaneously. Prior work using the TCGA-HNSC dataset has focused primarily on addressing missingness and high dimensionality in combined clinical-genomic feature sets to predict recurrence-free survival [6]. A recent systematic review of structured-data ML models in head and neck cancer similarly found that ML approaches frequently outperformed conventional Cox regression in time-to-event analyses. Still, methodological reporting and risk of bias varied substantially across the available literature [7]. Empirical comparisons of classifier families across multiple radiotherapy cohorts, including head and neck cancer, have shown that random forest, support vector machine, and gradient boosting typically achieve similar discrimination on clinical data [8]. However, many published pipelines mix real computation with unclear reporting of cross-validation procedures, and relatively few studies report transparent, fully reproducible clinical-only baselines against which more complex multi-omic models can be benchmarked.

The present study had three aims: (i) to build and rigorously cross-validate ML classifiers that predict vital status at last contact in HNSCC, using only routinely available clinicopathological variables from the TCGA-HNSC cohort; (ii) to complement this classification approach with formal time-to-event analysis (Kaplan-Meier and Cox regression), which properly accounts for right-censoring a source of bias that binary classification alone cannot address; and (iii) to test, in the subset of patients with available tumour RNA-sequencing data, whether incorporating a small panel of known HNSCC driver genes and an unsupervised transcriptomic signature improves discrimination over the clinicopathological baseline. This dual, and then extended, approach is intended to provide an honest, clinically interpretable prognostic baseline rather than an inflated performance claim.

## Materials and methods

Data source and cohort

Clinical data for 528 patients diagnosed with HNSCC were obtained from the TCGA-HNSC project, as curated and distributed via the National Cancer Institute's (NCI's) Genomic Data Commons (GDC) clinical data schema [5, 9]. Data were accessed as a de-identified, publicly available clinical data file; no patient-identifiable information was used, and no additional ethical approval was required beyond the original TCGA consent and data-access framework.

Feature selection

Ten clinicopathological variables available for the majority of patients were selected a priori for modelling: age at diagnosis, sex, self-reported race, AJCC pathologic stage (collapsed to Stage I-IV, with Stage IVA/IVB/IVC combined given small subgroup sizes), pathologic T and N classification, primary tumour subsite (grouped into oral cavity, oropharynx, larynx, hypopharynx, and overlapping/not-otherwise-specified categories), alcohol use history, prior malignancy, and receipt of radiation therapy or systemic (pharmacological) therapy. Tumour grade was recorded as "not reported" for the entire cohort and was excluded. Pack-years smoked were missing for 43.6% of patients and were excluded from the primary model to avoid excessive imputation-driven bias, consistent with guidance that imputation of variables with very high missingness can introduce more bias than it resolves [10]; alcohol history and smoking-related exposure are discussed as limitations below.

Outcome definitions

The primary classification outcome was vital status at last contact (deceased vs. alive), coded from the GDC demographic field. For time-to-event analysis, survival time was defined as days from diagnosis to death (for deceased patients) or to last follow-up (for censored patients); 526 of 528 patients had a valid non-negative survival time and were included in the survival analysis.

Statistical and ML methods

All analyses were performed in Python 3 using scikit-learn (v1.x; Python Software Foundation, Wilmington, DE, USA) for ML [11] and lifelines for survival analysis [12]. Categorical variables were one-hot encoded, and missing categorical values were encoded as an explicit "Missing" category rather than being silently dropped, since missingness in variables such as radiation treatment status was itself informative. The continuous age variable was median-imputed (one missing value) and standardised. Four classifiers were trained: logistic regression (L2-regularised, class-balanced), random forest (500 trees, maximum depth 5, class-balanced), gradient boosting (200 estimators, maximum depth 3, learning rate 0.05), and a support vector machine with radial basis function kernel (class-balanced). All preprocessing steps (imputation, encoding, scaling) were embedded inside a single scikit-learn pipeline together with the classifier and refit within each cross-validation fold, preventing information leakage from the held-out fold into training. Model discrimination was evaluated by stratified five-fold cross-validation, reporting the area under the receiver operating characteristic curve (AUC-ROC), accuracy, precision, recall, and F1-score at a 0.5 probability threshold, along with the confusion matrix. Feature importance was extracted from the gradient boosting model fitted on the full dataset.

For survival analysis, Kaplan-Meier curves were estimated separately for each AJCC stage group, with between-group comparison for Stage I vs. Stage IV using the log-rank test. A multivariable Cox proportional hazards model was fitted with age, AJCC stage, and sex as covariates; model discrimination was summarised using Harrell's concordance index.

Genomic feature integration

To assess whether tumour transcriptomic data add prognostic value beyond clinicopathological variables, we obtained a curated RNA-sequencing expression panel for the TCGA-HNSC cohort (4,465 genes profiled across 544 aliquots), sourced from the same GDC-derived public compilation as the clinical data [9]. After restricting to primary tumour aliquots (sample type code '01') and excluding solid-tissue-normal samples, 500 patients had a unique tumour expression profile; all 500 also had corresponding clinicopathological records, yielding a matched clinical-plus-genomic analytic cohort of 500 patients (218 deaths, 43.6%), slightly smaller than the full 528-patient clinical cohort.

Raw counts were normalised for sequencing depth using counts-per-million (CPM) scaling, consistent with standard RNA-seq normalisation practice [13], and log2(CPM+1)-transformed. From an a priori list of 15 genes reported as recurrently altered in the TCGA-HNSC landmark analysis [5], TP53, PIK3CA, NOTCH1, CDKN2A, EGFR, CASP8, HRAS, NSD1, FAT1, TRAF3, E2F1, CCND1, MYC, NFE2L2, and AJUBA only four (CDKN2A, EGFR, FAT1, and E2F1) were present within this reduced 4,465-gene panel and were retained as individual driver-gene expression features. To additionally capture broader, unsupervised transcriptomic structure without a full differential-expression analysis, the panel-wide log-CPM matrix was reduced to its leading principal components (PC1-PC5), following the precedent of unsupervised dimensionality reduction applied to this same dataset by Rendleman et al. [6]. As with the clinicopathological pipeline under the statistical and machine learning methods section, standardisation and PCA were fitted only on each cross-validation training partition, with held-out-fold expression values then projected onto those training-derived components so that no information from a held-out patient contributed to the principal-component axes used to predict that patient. For descriptive purposes only, the same five components fitted once on the full panel-wide matrix (all 500 patients) explained 32.8% of total expression variance.

The four driver-gene expression values and five principal components were combined with the 10 clinicopathological variables described in the feature selection section to form an expanded "clinical + genomic" feature set. The same four classifiers, preprocessing pipeline, and stratified five-fold cross-validation procedure described under the statistical and ML methods section were reapplied to this 500-patient matched cohort twice: once using clinicopathological variables alone (for a like-for-like comparison against the genomic-augmented model) and once using the expanded clinical + genomic feature set. Feature importance for the augmented model was again extracted from a gradient boosting model fitted on the full matched cohort.

## Results

Cohort characteristics

Table 1 summarises the baseline characteristics of the 528-patient cohort. Median age at diagnosis was 61 years (range 19-90 years); 73.1% of patients were male, consistent with the well-established male predominance of HNSCC. The majority of patients presented with locally advanced disease: 51.1% had Stage IV disease at diagnosis and only 5.1% presented with Stage I disease. Overall, 224 patients (42.4%) were recorded as deceased at last contact.

**Table 1:** Baseline characteristics of the TCGA-HNSC analytic cohort (n = 528). AJCC: American Joint Committee on Cancer; TCGA-HNSC: The Cancer Genome Atlas Head-Neck Squamous Cell Carcinoma

Characteristic	Category	n (%) or Mean ± SD
Total cohort		528 (100)
Age at diagnosis (years)	Mean ± SD (range)	60.9 ± 11.9 (19–90)
Sex	Male	386 (73.1)
	Female	142 (26.9)
Race	White	452 (85.6)
	Black or African American	48 (9.1)
	Asian	11 (2.1)
	American Indian/Alaska Native	2 (0.4)
	Not reported	15 (2.8)
Alcohol use history	Yes	352 (66.7)
	No	165 (31.3)
	Not reported	11 (2.1)
AJCC pathologic stage	I	27 (5.1)
	II	74 (14.0)
	III	82 (15.5)
	IV (A/B/C combined)	270 (51.1)
	Missing	75 (14.2)
Prior malignancy	Yes	27 (5.1)
	No	501 (94.9)
Radiation therapy	Yes	325 (61.6)
	No	143 (27.1)
	Not reported	60 (11.4)
Pharmacological (systemic) therapy	Yes	189 (35.8)
	No	275 (52.1)
	Not reported	64 (12.1)
Vital status at last contact	Deceased	224 (42.4)
	Alive	304 (57.6)

Classification model performance

Table 2 and Figure 1 summarise cross-validated performance for the four classifiers predicting vital status. Random forest achieved the highest AUC-ROC (0.698), narrowly ahead of logistic regression (0.689) and support vector machine (0.686); gradient boosting performed slightly worse (0.666). Accuracy was similar across models (0.634-0.648). Logistic regression achieved the best recall (0.607) at the cost of more false-positive predictions, reflecting its class-balanced weighting, whereas tree-based models and support vector machine traded recall for modestly higher precision. No model exceeded an AUC of 0.70, indicating that clinicopathological variables alone provide real but limited discriminatory power for individual-level mortality prediction.

**Table 2:** Five-fold cross-validated performance of four machine learning classifiers for mortality prediction (n = 528). AUC-ROC: area under the receiver operating characteristic curve; SVM: support vector machine; RBF: radial basis function

Model	AUC-ROC	Accuracy	Precision	Recall (Sensitivity)	F1-score
Logistic Regression	0.689	0.648	0.581	0.607	0.594
Random Forest	0.698	0.644	0.595	0.504	0.546
Gradient Boosting	0.666	0.636	0.590	0.469	0.522
SVM (RBF Kernel)	0.686	0.634	0.589	0.460	0.516

**Figure 1:**
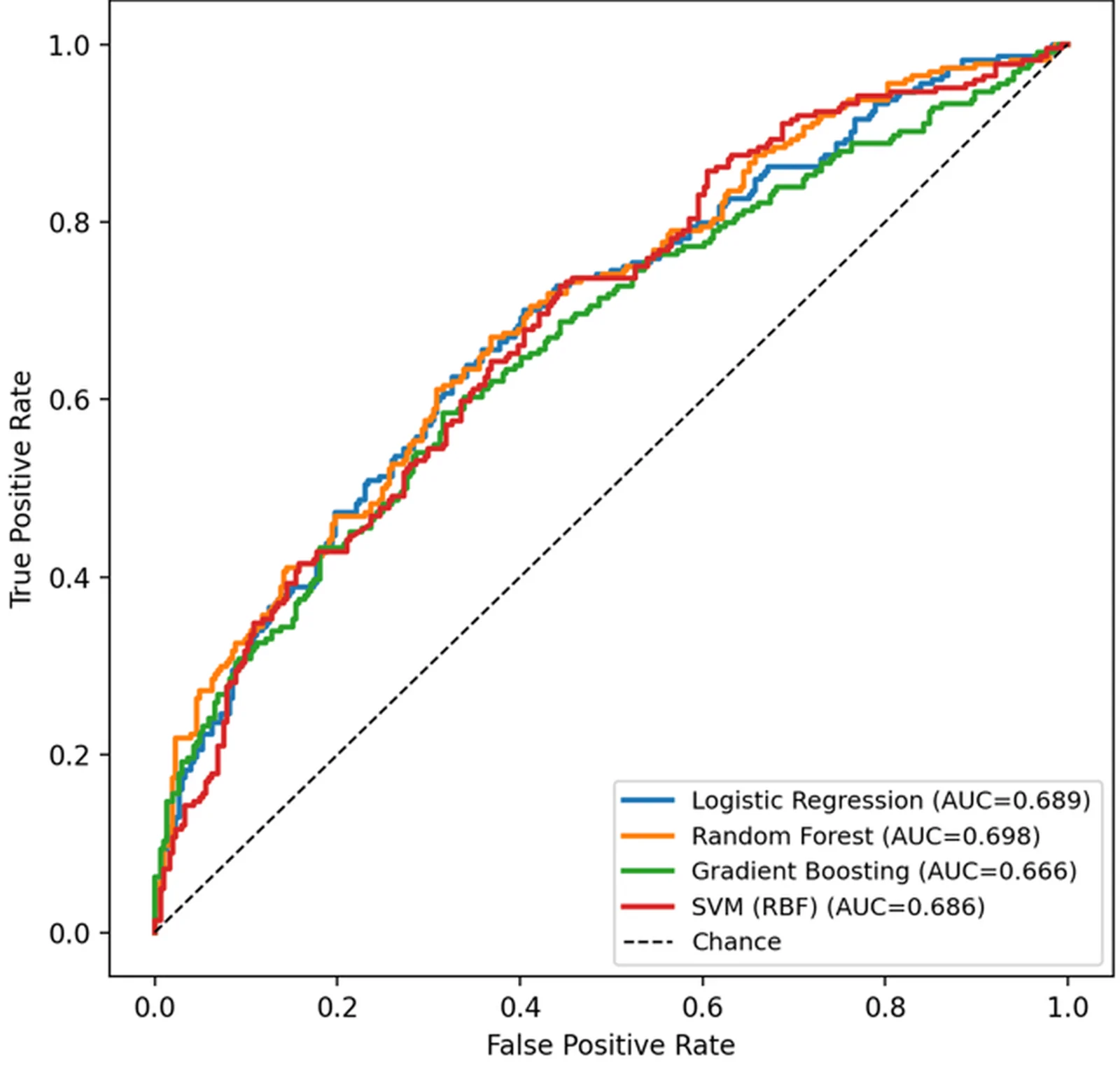
Receiver operating characteristic (ROC) curves for four classifiers predicting vital status, derived from stratified five-fold cross-validation (n = 528). AUC: area under the curve; SVM: support vector machine; RBF: radial basis function

Predictor importance

Feature importance derived from the gradient boosting model (Figure 2) identified age at diagnosis as the dominant predictor (relative importance 0.277), followed by missingness of radiation treatment status (0.104, itself a marker of incomplete follow-up or non-standard care pathway), node-negative (N0) status (0.067), and Stage IV disease (0.032). Race (Black or African American vs. White) contributed a small but non-negligible importance (0.025), consistent with previously reported outcome disparities in HNSCC that merit further investigation with adjustment for access-to-care variables not captured in this dataset.

**Figure 2:**
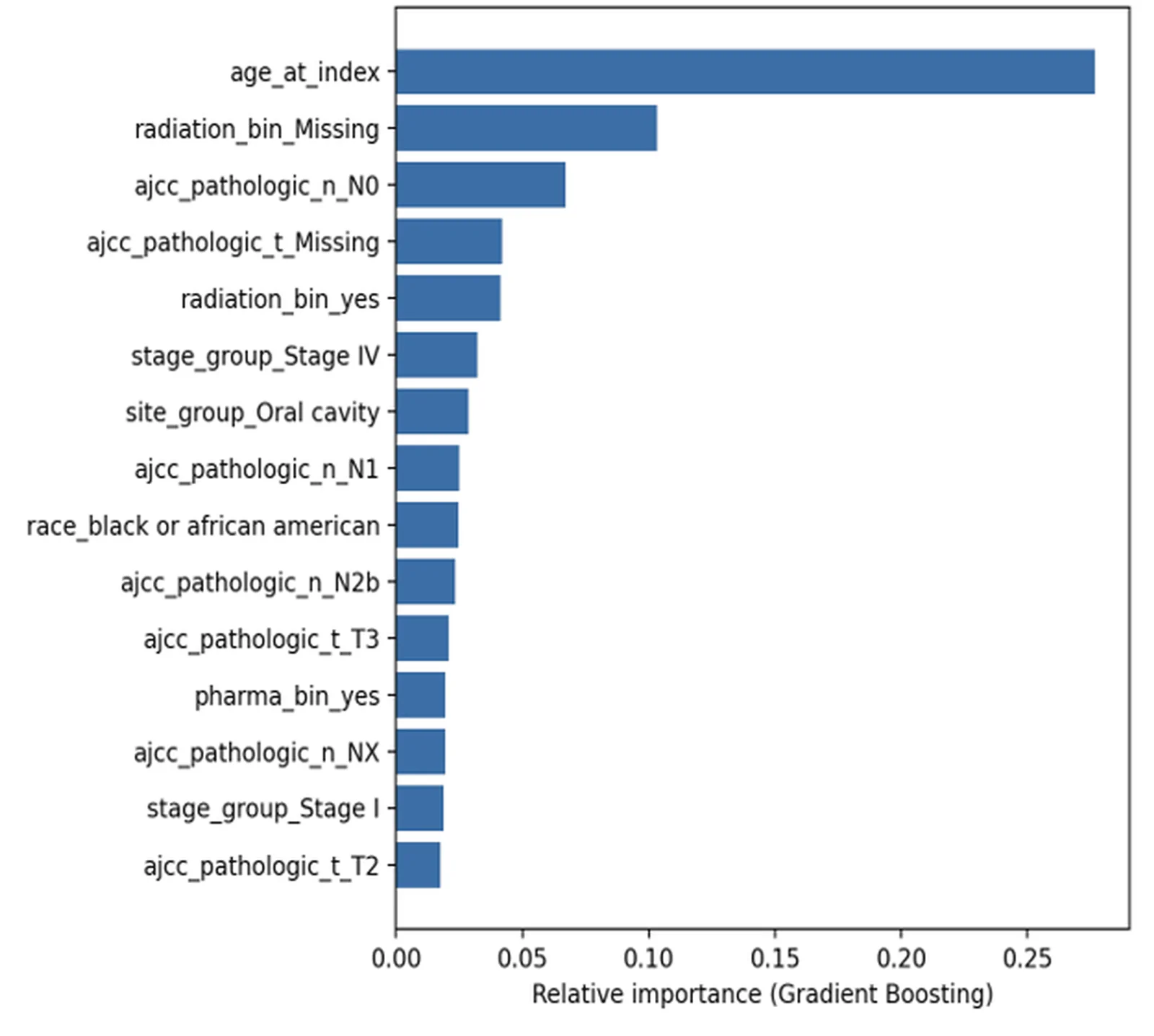
Top clinicopathological predictors of mortality, ranked by relative feature importance from the gradient boosting model (n = 528).

Survival analysis

Of 528 patients, 526 had valid survival-time data (223 deaths). Kaplan-Meier estimates (Figure 3) showed a clear, clinically expected gradient in overall survival by AJCC stage, with Stage I patients showing markedly better survival than Stage III-IV patients (log-rank test, Stage I vs. Stage IV, p = 0.005).

**Figure 3:**
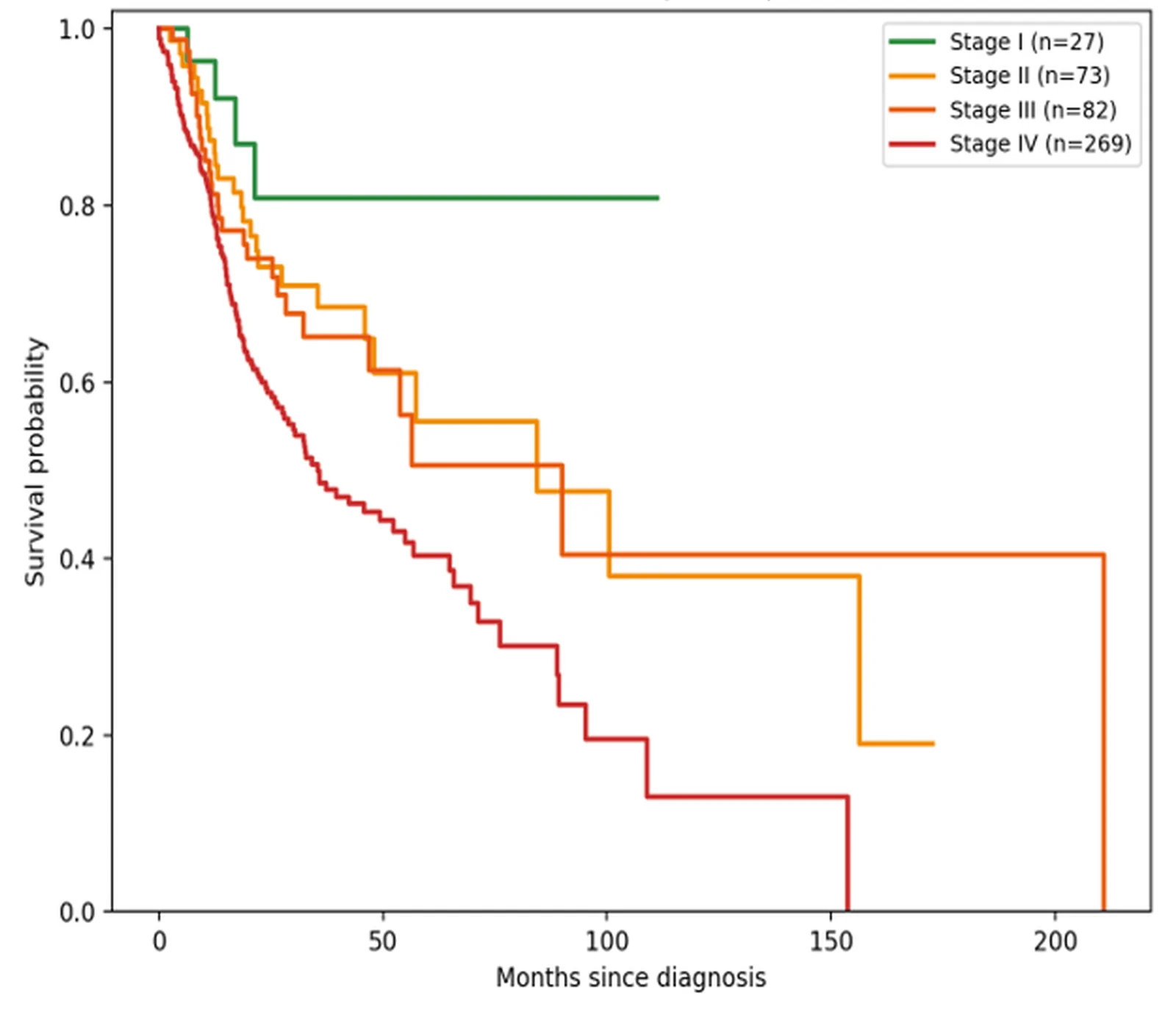
Kaplan-Meier overall survival curves stratified by AJCC pathologic stage (n = 526). AJCC: American Joint Committee on Cancer

Multivariable Cox regression (Table 3) confirmed that Stage IV disease was independently associated with more than a four-fold increase in mortality hazard relative to Stage I (HR 4.07, 95% CI 1.50-11.03, p = 0.006) and that each additional year of age increased mortality hazard by 2% (HR 1.02, 95% CI 1.01-1.03, p = 0.005). The association between male sex and lower hazard did not reach statistical significance (HR 0.77, 95% CI 0.56-1.05, p = 0.100). The model's concordance index was 0.608, indicating modest discriminative ability consistent with the classification results above.

**Table 3:** Multivariable Cox proportional hazards model for overall survival (n = 451 with complete covariate data).

Covariate	Hazard Ratio (95% CI)	p-value
Age at diagnosis (per year)	1.02 (1.01–1.03)	0.005
Stage II vs Stage I	2.04 (0.71–5.86)	0.185
Stage III vs Stage I	2.31 (0.81–6.61)	0.118
Stage IV vs Stage I	4.07 (1.50–11.03)	0.006
Male vs Female	0.77 (0.56–1.05)	0.100

Effect of genomic feature integration

Restricting to the 500 patients with matched RNA-sequencing data reproduced the clinical-only performance pattern seen in the full cohort (Table 4; dashed curves in Figure 4): AUC ranged from 0.666 (gradient boosting) to 0.708 (random forest). Adding the four driver-gene expression features and five transcriptomic principal components improved discrimination for every classifier (Table 4; solid curves in Figure 4), with the largest gains for random forest (AUC 0.708 → 0.727) and logistic regression (AUC 0.688 → 0.704); gradient boosting improved only marginally (0.666 → 0.676). Accuracy, precision, and recall improved concordantly for the two best-performing models.

**Table 4:** Five-fold cross-validated classifier performance with and without genomic features, matched cohort (n = 500). AUC: area under the curve; SVM: support vector machine; RBF: radial basis function

Model	AUC (Clinical-only)	AUC (Clinical+Genomic)	Δ AUC	Accuracy (Clinical+Genomic)	F1-score (Clinical+Genomic)
Logistic Regression	0.688	0.704	+0.016	0.666	0.635
Random Forest	0.708	0.727	+0.019	0.688	0.642
Gradient Boosting	0.666	0.676	+0.010	0.638	0.560
SVM (RBF Kernel)	0.696	0.713	+0.017	0.662	0.585

**Figure 4:**
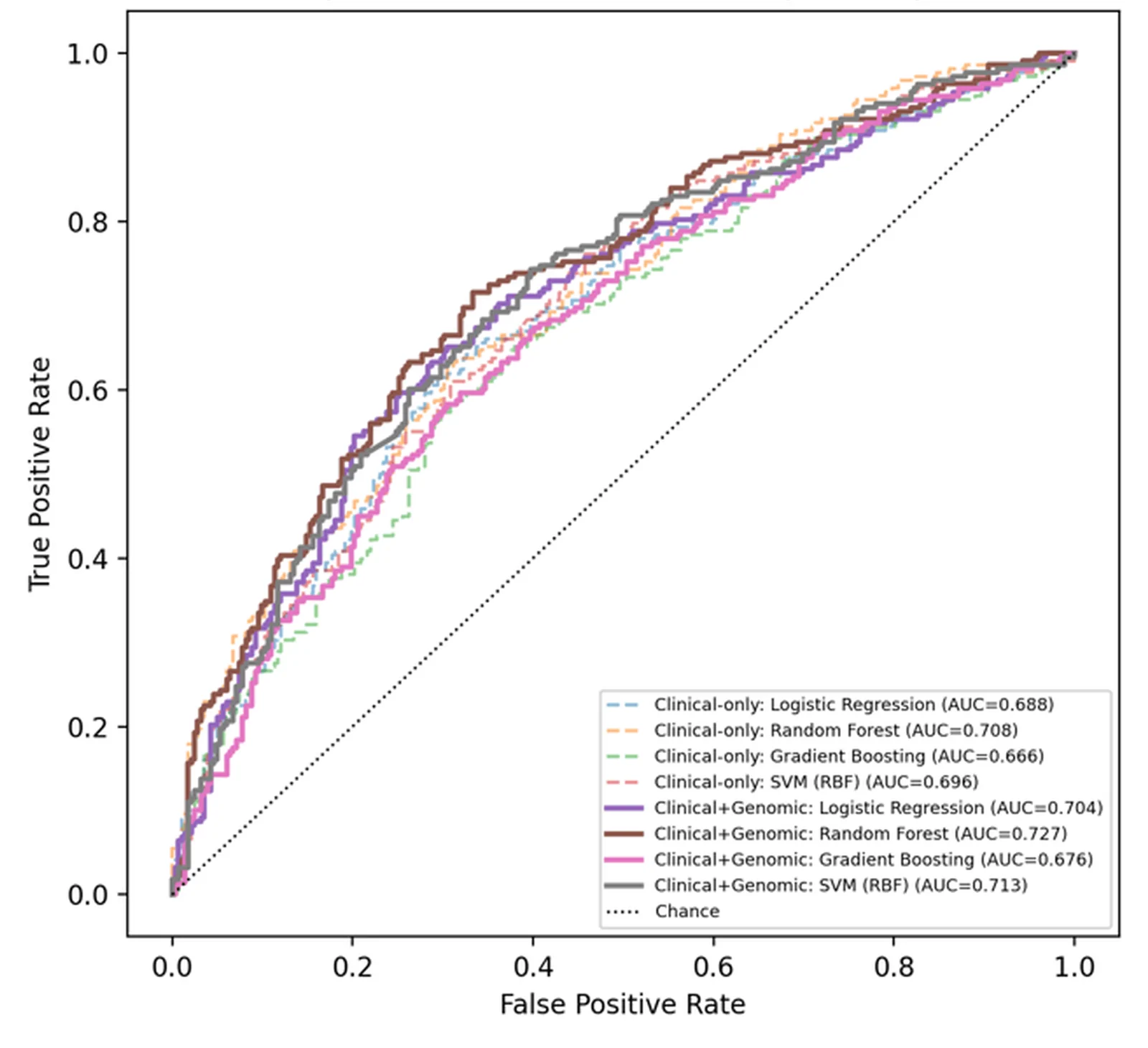
Receiver operating characteristic (ROC) curves comparing clinical-only (dashed) and clinical+genomic (solid) models across four classifiers, matched cohort (n = 500). AUC: area under the curve; SVM: support vector machine; RBF: radial basis function

Genomic predictor importance

Feature importance from the gradient boosting model fitted on the expanded feature set (Figure 5) showed that genomic features occupied eight of the fifteen most important predictors, led by the fifth transcriptomic principal component (PC5, relative importance 0.110) and CDKN2A expression (0.090), each ranking above or comparably to age at diagnosis (0.099) and AJCC nodal status (N0, 0.064). FAT1 (0.084), PC3 (0.074), PC1 (0.073), PC2 (0.057), EGFR (0.054), and E2F1 (0.033) expression collectively accounted for a further substantial share of total importance, indicating that transcriptomic information contributed materially, rather than marginally, to model discrimination in this subset.

**Figure 5:**
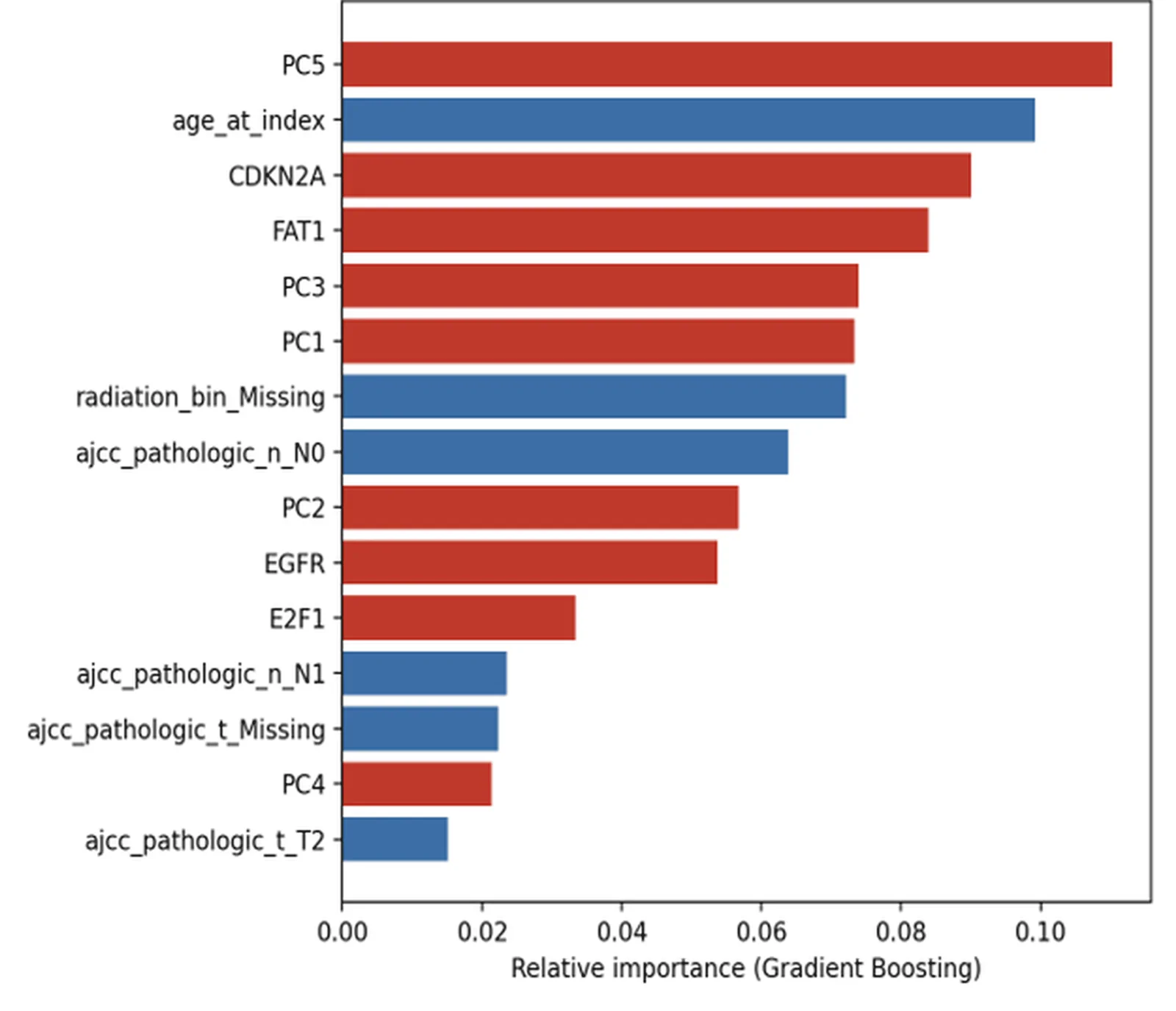
Top predictors of mortality in the clinical+genomic gradient boosting model; genomic features shown in red (n = 500).

## Discussion

This analysis demonstrates that routinely collected clinicopathological variables carry statistically robust, but individually modest, prognostic information in HNSCC. Across four independent ML algorithms, AUC values clustered tightly between 0.666 and 0.698, a range that is unlikely to reflect algorithm choice and is more likely to reflect a genuine ceiling on the prognostic content of clinical variables alone in this cohort, a pattern consistent with empirical comparisons showing that classifier family choice explains relatively little of the variance in discrimination once a stable set of clinical predictors is fixed [8]. This is an important, and often under-reported, finding: many published HNSCC prediction studies emphasise the single best-performing model without contextualising how close the alternatives perform, potentially overstating the added value of algorithmic sophistication over simpler baselines such as logistic regression.

The identification of age, nodal status, and AJCC stage as leading predictors is biologically expected and corroborated by the independent Cox regression, which found stage and age to be significant, statistically robust predictors of survival even after accounting for right-censoring, echoing an independent Surveillance, Epidemiology, and End Results (SEER) Program-based comparison of tree-based ML algorithms and Cox regression in oral and pharyngeal cancers that likewise identified age and stage as the dominant prognostic variables regardless of modelling approach [14]. The convergence between the tree-based feature importance ranking (age, node status, stage) and the Cox hazard ratios (age, stage) strengthens confidence that these associations are not artefacts of a single modelling approach.

These findings should be read alongside the broader literature on ML applications to the TCGA-HNSC dataset. Rendleman et al. addressed sparsity and high dimensionality in combined clinical and RNA expression data from more than 500 TCGA-HNSC cases and reported that imputation improved the prognostic utility of sparse clinical treatment variables for predicting two-year recurrence-free survival [6]. Our results are consistent with their broader conclusion that TCGA-HNSC clinical fields are informative but substantially limited by missingness and extend this by providing a fully cross-validated, leakage-controlled comparison across four classifier families with an explicit survival-analysis complement. This is also broadly concordant with a recent clinical-data-only mortality and relapse-free survival model in HNSCC, which similarly relied on routinely captured variables such as locoregional control and treatment type rather than molecular features to achieve clinically meaningful discrimination [15], and with the systematic review noted above, which found that clinicopathological ML models for HNC are common in the literature but are inconsistently benchmarked against transparent baselines [7].

Incorporating even a coarse, panel-limited transcriptomic signature of four driver genes plus five unsupervised principal components produced a consistent, if modest, improvement in discrimination across every classifier tested (ΔAUC +0.010 to +0.019), with the largest gain for random forest (AUC 0.727). This is congruent with Rendleman et al.'s finding that transcriptomic and imputed clinical data together improved prognostic modelling relative to clinical variables alone in this same cohort [6] and suggests that the discrimination ceiling described above is not fixed at the clinical level but can be modestly extended, without yet being resolved, by adding molecular data. The prominence of CDKN2A and EGFR expression is biologically plausible: CDKN2A (p16) inactivation and EGFR pathway activity are both central, well-characterised events in HNSCC pathogenesis captured in the original TCGA genomic landscape analysis [5], and CDKN2A/p16 expression is additionally used clinically as a surrogate marker for HPV-driven oropharyngeal disease, which independently predicts favourable prognosis [16]. However, the magnitude of improvement was small relative to the effort and data requirements of genomic profiling, echoing the broader observation that structured-data machine learning models for HNC, whether clinical, genomic, or combined, remain inconsistently benchmarked against transparent baselines in the published literature [7].

The modest importance attributable to race in our gradient boosting model, together with the well-documented global disparities in HNSCC incidence and mortality by socioeconomic development level [1,2], and with population-based U.S. data showing that Black patients with HNSCC present with more advanced stages and experience worse overall survival than White patients independent of many clinical covariates [17], suggests that future clinicopathological models should explicitly incorporate access-to-care and socioeconomic variables, which were not available in this dataset, before race-related associations are interpreted as biological rather than structural.

Several limitations should be considered. First, this is a retrospective, single-consortium cohort; TCGA sites are predominantly large academic medical centres in high-income settings, limiting generalisability to broader, more heterogeneous patient populations. Second, key prognostic variables, including HPV/p16 status, tumour grade, and quantitative smoking exposure (pack-years), were unavailable or too sparsely populated for reliable inclusion; HPV status in particular is a well-established major determinant of oropharyngeal HNSCC prognosis, with landmark cohort data showing markedly better survival in HPV-positive versus HPV-negative disease independent of stage and treatment intensity [15], and its absence likely constrains achievable model performance. Third, the binary classification approach does not account for length of follow-up and is therefore complementary to, rather than a replacement for, the Cox survival analysis reported alongside it. Fourth, no external validation cohort was available; reported performance should be interpreted as internal cross-validated estimates rather than evidence of generalisable clinical utility [4].

Fifth, the genomic extension in the effect of genomic feature integration section relied on a pre-curated 4,465-gene expression panel rather than the whole transcriptome, was normalised using simple library-size (CPM) scaling rather than a full RNA-seq differential-expression pipeline with batch correction (e.g., TMM or DESeq2-style methods) [13], and only four of 15 a priori candidate driver genes from the TCGA landmark analysis [5] were represented in this panel; a whole-transcriptome analysis with proper differential expression testing and multiple-testing correction would likely identify additional informative genes and yield a more complete transcriptomic signature. Sixth, the matched clinical+genomic cohort (n = 500) excluded 28 patients without RNA-sequencing data and is not identical in composition to the full 528-patient clinical cohort, so the clinical-only and genomic-augmented analyses, while directionally consistent, are not perfectly nested. Finally, tumour mutation data, copy-number profiles, HPV/p16 status, and radiomic features, each shown elsewhere to add independent prognostic value, remain natural directions for future multimodal extensions of this pipeline.

## Conclusions

Using a transparent, leakage-controlled ML pipeline and a complementary formal survival analysis, this study shows that clinicopathological variables in the TCGA-HNSC cohort provide real, statistically significant, but individually modest prognostic value for mortality risk in HNSCC. Age and AJCC stage emerged as the most robust and consistent predictors across both ML and classical survival modelling frameworks. In a matched subset with available RNA-sequencing data, supplementing clinicopathological variables with a small panel of driver-gene expression values and an unsupervised transcriptomic signature further improved discrimination, without eliminating the underlying prognostic ceiling. These results support the use of routinely available clinical data as an interpretable prognostic baseline, while reinforcing that stepwise, transparently benchmarked multi-omic integration rather than reliance on any single data modality is the more realistic path toward clinically useful individual-level risk prediction in HNSCC.
